# Relationship of FDG Uptake of the Reticuloendothelial System with Tumor Immune Microenvironment and Prognosis in Patients with Gastric Cancer

**DOI:** 10.3390/life13030771

**Published:** 2023-03-13

**Authors:** Hyein Ahn, Geum Jong Song, Moon-Soo Lee, Ji-Hye Lee, Si-Hyong Jang, Mee-Hye Oh, Jong Hyuk Yun, Sang Mi Lee, Jeong Won Lee

**Affiliations:** 1 Department of Pathology, Soonchunhyang University Cheonan Hospital, 31 Suncheonhyang 6-gil, Dongnam-gu, Cheonan 31151, Republic of Korea; 2 Department of Surgery, Soonchunhyang University Cheonan Hospital, 31 Suncheonhyang 6-gil, Dongnam-gu, Cheonan 31151, Republic of Korea; 3 Department of Nuclear Medicine, Soonchunhyang University Cheonan Hospital, 31 Suncheonhyang 6-gil, Dongnam-gu, Cheonan 31151, Republic of Korea; 4 Department of Nuclear Medicine, College of Medicine, Catholic Kwandong University, International St. Mary’s Hospital, 25 Simgok-ro 100-gil, Seo-gu, Incheon 22711, Republic of Korea

**Keywords:** bone marrow, F-18 fluorodeoxyglucose, positron emission tomography, prognosis, spleen, stomach neoplasm

## Abstract

2-deoxy-2-[^18^F]fluoro-D-glucose (FDG) uptake of the reticuloendothelial system, including the bone marrow (BM) and spleen, on positron emission tomography/computed tomography (PET/CT) has been shown to be a significant prognostic factor in diverse malignancies. However, the relationship between FDG uptake of the BM and spleen and histopathological findings, including the tumor immune microenvironment, has not been fully evaluated. This study aimed to investigate the relationship of FDG uptake in the BM and spleen with histopathological findings and recurrence-free survival (RFS) in patients with gastric cancer. Seventy patients with gastric cancer who underwent pre-operative FDG PET/CT and subsequent curative surgery were retrospectively enrolled. On image analysis, the BM-to-liver uptake ratio (BLR) and spleen-to-liver uptake ratio (SLR) were measured from PET/CT images, and on immunohistochemical analysis, the densities of immune cell infiltration in the tumor tissue were graded. The BLR and SLR showed significant positive correlations with the grades of CD163 cell and CD8 cell infiltration in the tumor tissue, respectively (*p* < 0.05). In multivariate survival analysis, both BLR and SLR were significant predictors of RFS (*p* < 0.05). FDG uptake in the BM and spleen might be potential imaging biomarkers for evaluating tumor immune microenvironment conditions and predicting RFS in patients with gastric cancer.

## 1. Introduction

Cancer has long been recognized as having an intimate relationship with inflammation [1]. In recent decades, several studies have demonstrated that inflammation can involve every process of tumor development, progression, metastasis, and treatment resistance, suggesting that inflammation is one of the hallmarks of cancer [1,2,3]. Due to of the notable association of gastric carcinogenesis with *Helicobacter pylori* infection and Epstein–Barr virus infection, gastric cancer has been considered to be closely associated with infection and chronic inflammation [4]. In clinical studies of gastric cancer patients, serum inflammatory markers such as C-reactive protein, the neutrophil-to-lymphocyte ratio (NLR), and platelet-to-lymphocyte ratio (PLR), which can reflect the degree of host inflammatory response, were found to be significant predictors of cancer progression and survival [5,6]. Furthermore, recently, tumor immunotherapy using immune checkpoint inhibitors targeting programmed cell death-1 (PD-1) or programmed cell death ligand-1 (PD-L1) has been developed; therefore, interactions between cancer cells and immune cells in the tumor microenvironment have become a focus of attention in gastric cancer research [2,7]. In previous studies, immune cell infiltration in tumor tissue and metabolites and cytokines secreted by immune cells and cancer cells were found to critically affect progression and treatment failure in patients with gastric cancer [7,8,9].

Currently, 2-deoxy-2-[^18^F]fluoro-D-glucose (FDG) positron emission tomography/computed tomography (PET/CT) has shown clinical significance in detecting metastasis and predicting prognosis in patients with various malignant diseases, including gastric cancer [10,11,12]. As FDG is not a tumor-specific agent and can also be used to assess the glucose metabolic activity of the organ, several previous studies attempted to estimate the degree of systemic inflammatory response using FDG PET/CT images [13,14]. In a previous study, patients with malignant disease showed significantly increased FDG uptake in the bone marrow (BM) and spleen, which are the major organs of the reticuloendothelial system that play a crucial role in the host inflammatory response to cancer cells [15,16,17]. Moreover, a number of studies have demonstrated that FDG uptake in the BM and spleen showed significant correlations with serum inflammatory markers, including NLR and PLR, and patients with high FDG uptake in the BM and spleen had significantly worse clinical outcomes than those with low uptake in various kinds of malignant diseases [14,18,19,20]. Therefore, FDG uptake in the BM and spleen has been suggested as an imaging biomarker of PET/CT to estimate the host systemic inflammatory response to cancer cells [19,20]. However, until now, there are no studies that compared the relationship of FDG uptake of both BM and spleen with the tumor immune microenvironment in a single study [14,21,22,23]. Furthermore, only a few studies have evaluated the clinical value of FDG uptake of the reticuloendothelial system in patients with gastric cancer [24,25].

Thus, the present study aimed to investigate the relationship of FDG uptake in the BM and spleen on staging FDG PET/CT with the tumor immune microenvironment based on immunohistochemical analysis and recurrence-free survival (RFS) in patients with gastric cancer who underwent curative surgical resection.

## 2. Materials and Methods

### 2.1. Patients

We performed a retrospective review of the medical records of patients who were histopathologically diagnosed with gastric cancer and underwent FDG PET/CT for staging work-up between March 2014 and June 2022 at Soonchunhyang University Cheonan Hospital. Among them, a total of 70 patients who met the following inclusion criteria were enrolled in the present study: patients (1) who showed no distant metastasis on staging work-up imaging studies or peritoneal metastases on surgical exploration, (2) in whom staging PET/CT images and surgical specimens were available for analysis, and (3) who underwent surgery with curative intent. Patients who met the following criteria were excluded from the study: patients (1) who had a previous history of hematologic diseases, chronic liver diseases, or other malignant diseases, (2) who received neoadjuvant chemotherapy before the surgery or received palliative surgery, (3) who were diagnosed with active inflammatory disease at the time of PET/CT scan, and (4) who had a follow-up period of less than 12 months after the surgery. Along with FDG PET/CT, all enrolled patients underwent physical examination, blood tests, esophagogastroduodenoscopy, and contrast-enhanced abdominopelvic CT for staging work-up. Based on the results of these examinations, gastrectomy with regional lymph node dissection was performed, and adjuvant chemotherapy was recommended for patients with TNM stages II–III. The median interval between FDG PET/CT and surgery was 4 days (interquartile range; 1–10 days). After treatment, routine follow-up assessments, including blood tests, esophagogastroduodenoscopy, and contrast-enhanced abdominopelvic CT, were conducted every 6–8 months for the first 3 years and every 10–12 months thereafter.

### 2.2. FDG PET/CT Acquisition and Image Analysis

All enrolled patients were asked to fast for at least 6 h before the PET/CT scan. After confirmation of blood glucose level of <200 mg/dL, FDG dose of approximately 4.07 MBq/kg was intravenously injected. After 60 min of uptake period, PET/CT scans were performed using a hybrid PET/CT scanner (Biograph mCT128 scanner, Siemens Healthineers, Knoxville, TN, USA) from the skull base to the proximal thigh. A CT scan was first performed for attenuation correction using automatic dose modulation (100 mA and 120 kVp, slice thickness of 5 mm, and slice increment of 2.5 mm) without contrast enhancement. A PET scan was then performed for 1.5 min for the bed position in three-dimensional mode. PET images were reconstructed using an ordered subset expectation maximization reconstruction algorithm with attenuation correction, which involved 21 subsets and two iterations. The matrix size and slice thickness of the PET images were 128 × 128 and 5 mm, respectively. 

Two nuclear medicine physicians separately analyzed FDG PET/CT images of enrolled patients using OsiriX MD 10.0 software (Pixmeo, Geneva, Switzerland). For the quantitative PET/CT parameters, the average values of both reviewers’ measurements were recorded. Both reviewers were able to refer to the findings of esophagogastroduodenoscopy and other imaging studies but were unaware of other clinical information and follow-up results. To measure FDG uptake in gastric cancer lesions, the volume of interest (VOI) was manually drawn over the primary tumor lesion, and the highest value of FDG uptake, expressed as the standardized uptake value (SUV), in the VOI was calculated (tumor SUV). In cases with gastric cancer lesions that could not be distinguished from FDG uptake by the surrounding gastric wall uptake, the VOI of the gastric cancer lesion was drawn in accordance with the tumor location on esophagogastroduodenoscopy and contrast-enhanced CT images. FDG uptake in the liver, BM, and spleen was measured using a methodology described in previous studies [14,20,26]. A spherical 5 cm sized VOI and spherical 3 cm sized VOI were drawn in the right lobe of the liver and spleen, respectively, and the mean SUV of each VOI was defined as the liver SUV and spleen SUV, respectively. To measure FDG uptake in the BM, six spherical VOIs were constructed over the T10-T12 spines and L1-3 spines. Vertebrae that showed severe osteoarthritic change, compression fracture, or post-operative change were excluded from the BM uptake measurement. Using the cut-off value of 75% of the maximum SUV in each of the six VOIs, the mean SUV of the areas that had higher values of SUV than the cut-off values within the six VOIs was defined as the BM SUV. The spleen-to-liver uptake ratio (SLR) and BM-to-liver uptake ratio (BLR) were calculated using the liver SUV, spleen SUV, and BM SUV. Therefore, four PET/CT parameters of the BM and spleen, comprising the BM SUV, spleen SUV, BLR, and SLR, were measured for each patient.

### 2.3. Immunohistochemical Analysis 

Two experienced pathologists retrospectively conducted immunohistochemical analyses of surgical specimens of gastric cancer from the enrolled patients. Discrepancies between the two pathologists were resolved through consensus. Hematoxylin and eosin-stained slides of surgical specimens were reviewed, and cancer tissue areas that showed characteristic cellular morphology without necrotic areas were selected. The selected areas of each tissue block were cored twice using a 2 mm diameter cylinder and assembled into a recipient paraffin block using a tissue microarray instrument (Unitma, Seoul, Republic of Korea). Individual 4 µm thick slide sections were obtained from tissue microarray blocks and immunohistochemically stained using the Ventana Benchmark XT automated staining system (Ventana Medical Systems, Tucson, AZ, USA) according to the manufacturer’s protocol. Immunohistochemical staining for CD4, CD8, CD163, and interleukin-6 (IL-6) was performed using monoclonal rabbit anti-human CD4 (clone SP35, catalog number 7904423; Ventana Medical Systems), monoclonal mouse anti-human CD8 (clone C8/144B, catalog number IR623; Dako, Carpinteria, CA, USA), monoclonal mouse anti-human CD163 (clone OTI2G12, catalog number ab156769; Abcam, Cambridge, UK), and polyclonal rabbit anti-human IL-6 (catalog number ab6672; Abcam). Three representative areas were reviewed from each tissue microarray core under a high-power optical microscope at 400× magnification. The degree of CD4 cell, CD8 cell, and CD163 cell infiltration in the tumor tissue was graded according to the following criteria: grade 0, ≤10 cells; grade 1, 11–50 cells; grade 2, 51–100 cells; and grade 3, >100 cells. The degree of IL-6 expression in the tumor tissue was graded according to the following criteria: grade 0, negative; grade 1, focal light brown (weak); grade 2, light brown (moderate); and grade 3, brown (intense).

### 2.4. Statistical Analysis

The Mann–Whitney test was performed to compare FDG uptake in the BM and spleen between papillary/tubular types and mucinous/signet ring cells and other poorly cohesive types according to the World Health Organization (WHO) histopathological classification and between intestinal type and diffuse/indeterminate types according to the Lauren classification. The Kruskal–Wallis test was performed to assess the differences in the four BM and spleen parameters according to TNM stage, CD4, CD8, and CD163 cells infiltration grades, and IL-6 expression grade. A post hoc analysis using Dunn test was performed for the BM and spleen parameters, which revealed statistical significance on the Kruskal–Wallis test. Spearman correlation coefficients were calculated to evaluate the correlations between FDG uptake of the primary tumor, BM, and spleen. The primary outcome was RFS, defined as the time between the day of surgery and the detection of gastric cancer recurrence or death. Patients without recurrence or death were censored on the day of their last clinical follow-up. Univariate and multivariate Cox proportional hazard regression analyses were performed to evaluate the association between variables, including BM and spleen parameters and RFS, and hazard ratios with Wald 95% confidence intervals were calculated for each variable. Continuous variables included in the survival analysis were categorized into two groups according to the specific cut-off values, which were determined using receiver operating characteristic (ROC) curve analysis. Kaplan–Meier analysis was performed to estimate the cumulative RFS curves of BM and spleen parameters, and the log rank test was performed to evaluate RFS differences between patient groups. Statistical analyses were carried out using MedCalc Statistical Software version 20.210 (MedCalc Software Ltd., Ostend, Belgium). Results with a *p*-value < 0.05 were considered statistically significant.

## 3. Results

### 3.1. Patient Characteristics

**Figure 1:**
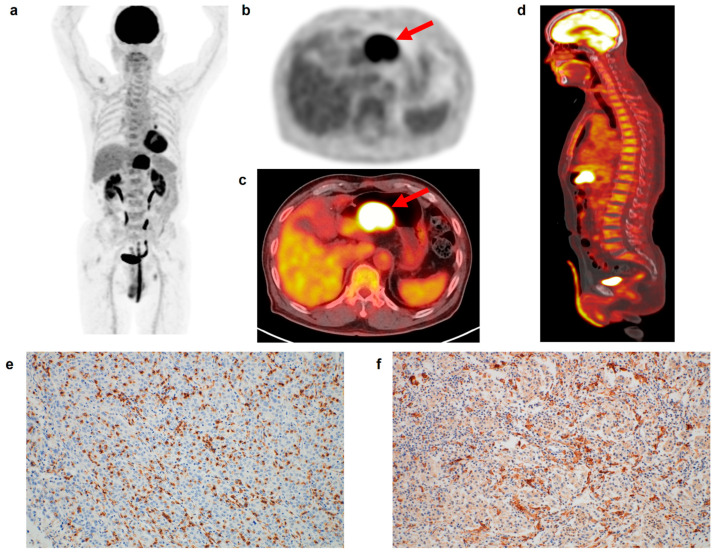
(**a**) Maximum intensity projection, (**b**) transaxial PET, and fused (**c**) transaxial and (**d**) sagittal FDG PET/CT images and immunohistochemical findings of (**e**) CD8 cell and (**f**) CD163 cell infiltrations in the tumor tissue of a 75-year-old man with gastric cancer. Intensely increased FDG uptake was found in the gastric cancer lesion (arrows in (**b**,**c**)) with tumor SUV of 24.6, and diffusely increased FDG uptake was observed in the BM and spleen, showing BLR of 1.05 and SLR of 1.01. The patient underwent total gastrectomy and was diagnosed with pT4N0 stage (TNM stage III). On immunohistochemical analysis, grade 3 of CD8 and CD163 cell infiltrations was found. The cancer recurred 22.6 months after the surgery.

**Figure 2:**
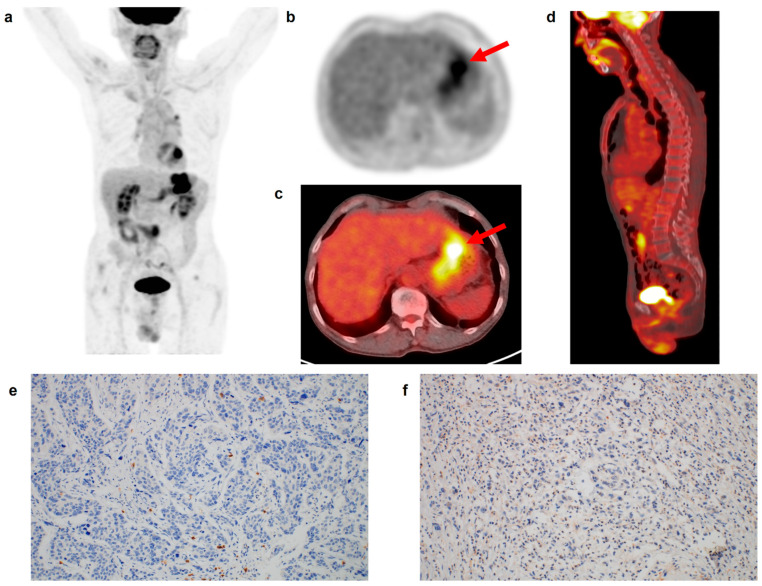
(**a**) Maximum intensity projection, (**b**) transaxial PET, and fused (**c**) transaxial and (**d**) sagittal FDG PET/CT images and immunohistochemical findings of (**e**) CD8 cell and (**f**) CD163 cell infiltrations in the tumor tissue of a 69-year-old man with gastric cancer. Intensely increased FDG uptake was found in the gastric cancer lesion (arrows in (**b**,**c**)) with tumor SUV of 15.7, and BLR and SLR were 0.75 and 0.76, respectively. The patient underwent total gastrectomy and was diagnosed with pT3N1 stage (TNM stage II). On immunohistochemical analysis, grade 0 of CD8 and CD163 cell infiltrations was found. During the follow-up period of 70.6 months, there was no event of recurrence or death in the patient.

The characteristics of the 70 patients with gastric cancer enrolled in this study are summarized in Table 1. On staging FDG PET/CT, 12 patients (17.1%) and 5 patients (7.1%) had higher FDG uptake in the BM (BLR > 1.00) and spleen (SLR > 1.00) than in the liver (Figure 1 and Figure 2). Of the five patients with an SLR > 1.00, four also showed a BLR > 1.00. On immunohistochemical analysis, 57.2% of the enrolled patients showed grade 2 or 3 infiltration of CD8 T cells, whereas the proportions of patients with grade 2 or 3 CD4 T cells and CD163 macrophages infiltrations and IL-6 expression were all less than 50.0%. The median RFS was 39.6 months (interquartile range, 16.2–70.7 months), and during the follow-up, 26 patients (37.1%) experienced cancer recurrence or death.

### 3.2. Correlation Analysis

**Figure 3:**
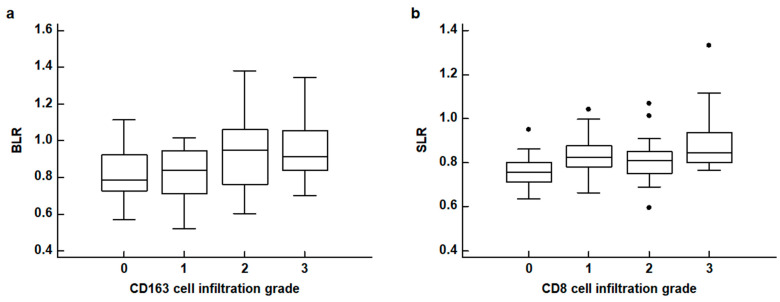
(**a**) Distribution of the bone marrow-to-liver uptake ratio (BLR) according to CD163 cell infiltration grades. (**b**) Distribution of the spleen-to-liver uptake ratio (SLR) according to CD8 cell infiltration grades (black dot: an outside value which is larger than 75 percentile value plus 1.5 times the interquartile range).

The results of the correlation analyses between FDG uptake of the BM and spleen and the histopathological results of gastric cancer are shown in Table 2. None of the four PET parameters of the BM and spleen revealed any significant relationship with the WHO classification and Lauren classification of histopathology (*p* > 0.05). For TNM stages, patients with advanced stages tended to show increased BLR and SLR with borderline significance (*p* = 0.094 for BLR and *p* = 0.080 for SLR). The BLR and SLR were significantly associated with the densities of CD163 cell infiltration and CD8 cell infiltration in the tumor tissue, respectively (*p* = 0.032 for BLR and *p* = 0.016 for SLR). None of the four PET parameters were significantly associated with the density of CD4 cell infiltration and IL-6 expression (*p* > 0.05). In post hoc analysis using the Dunn test, patients with grade 2 and 3 CD163 cell infiltration showed significantly higher BLR values than those with grade 0, and patients with grade 3 CD8 cell infiltration showed significantly higher SLR values than those with grade 0 (*p* < 0.05; Figure 3). 

The relationship between FDG uptake of the primary tumor, BM, and spleen is shown in Table S1. All four PET parameters of the BM and spleen were significantly positively correlated with tumor SUV (*p* < 0.05). Furthermore, except for the correlation between spleen SUV and BLR, all four PET parameters of the BM and spleen revealed significant positive correlations (*p* < 0.05).

### 3.3. Survival Analysis for RFS

The association of FDG PET/CT parameters of the BM and spleen with RFS was evaluated using the Cox proportional hazards regression model along with clinicopathological factors and tumor SUV. For the survival analysis, continuous variables were categorized into two groups based on the specific cut-off values on ROC curve analysis: age, 65 years; tumor SUV, 7.53; BM SUV, 1.64; spleen SUV, 1.67; BLR, 0.88; and SLR, 0.84. On univariate analysis, all four PET/CT parameters of the BM and spleen showed significant associations with RFS, showing worse RFS in patients with high parameter values (*p* < 0.05 for all; Table 3). In addition, T stage, N stage, TNM stage, adjuvant chemotherapy, CD163 cell infiltration, IL-6 expression, and tumor SUV were significant prognostic factors for RFS (*p* < 0.05). Patients with grade 3 CD8 cell infiltration also showed worse RFS than those with grade 0 infiltration, with borderline statistical significance (*p* = 0.087). 

**Figure 4:**
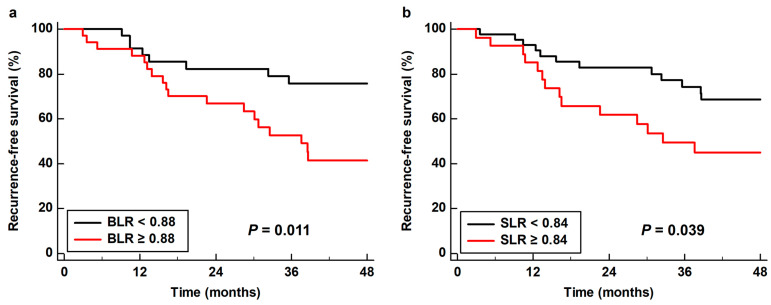
Cumulative recurrence-free survival curves based on (**a**) the bone marrow-to-liver uptake ratio (BLR) and (**b**) the spleen-to-liver uptake ratio (SLR).

As all four PET/CT parameters of the BM and spleen demonstrated statistical significance in univariate analysis, all of these parameters were further assessed using multivariate analysis. Considering the number of patients with events, only age, sex, TNM stage, and tumor SUV were included as covariates in the multivariate models. Moreover, because of the significant correlations between the PET/CT parameters of the BM and spleen (Table S1), the prognostic values of the BM SUV, spleen SUV, BLR, and SLR were assessed using separate models. Among the four parameters, BLR (*p* = 0.022; hazard ratio, 3.55; 95% confidence interval, 1.20–10.54) and SLR (*p* = 0.049; hazard ratio, 2.27; 95% confidence interval, 1.01–5.13) were significant predictors of RFS after adjusting for age, sex, TNM stage, and tumor SUV (Table 4). Kaplan–Meier analysis with log rank test showed that patients with high BLR and SLR showed significantly worse RFS than those with low BLR (*p* = 0.011) and SLR (*p* = 0.039). The 3-year RFS rates were 52.7 and 49.4% for patients with BLR ≥ 0.88 and SLR ≥ 0.84, respectively, whereas the 3-year RFS rates for patients with BLR < 0.88 and SLR < 0.84 were 75.8 and 74.3%, respectively (Figure 4). 

The combination of TNM stage and FDG uptake in the BM and spleen allowed further stratification of the recurrence risk in the enrolled patients (Table 5). Among patients with TNM stage I–II, there was no recurrence (0.0%) in patients with BLR < 0.88 and/or SLR < 0.84, while 42.9% of patients with both BLR ≥ 0.88 and SLR ≥ 0.84 experienced cancer recurrence. For patients with TNM stage III, up to 81.8% of patients with both BLR ≥ 0.88 and SLR ≥ 0.84 experienced cancer recurrence during follow-up.

## 4. Discussion

In previous studies, patients with malignant diseases showed significantly higher FDG uptake of the reticuloendothelial system than normal subjects or those with benign lung nodules [15,27]. This finding prompted several studies to investigate the underlying reasons for increased FDG uptake in the BM and spleen of patients with malignant diseases. For BM, the degree of FDG uptake is considered to be mainly correlated with hyperplasia of myeloid cells in the BM [28,29]. In a recent study performed in patients with uterine cervical cancer, patients with high FDG uptake in the BM revealed increased numbers of myeloid-derived suppressor cells in the tumor tissue and blood when compared with those with low uptake [23]. For the spleen, in another study of uterine cervical cancer patients, the density of diverse immune cells, including CD3, CD4, CD8, CD20, CD68, and CD163 cells, was numerically higher in a patient group with high spleen FDG uptake than a patient group with low uptake [14]. Moreover, a previous study of patients with hepatobiliary cancers revealed that FDG uptake in the spleen was significantly positively correlated with serum levels of pro-inflammatory cytokines, including IL-6 [30]. 

In the present study, we measured four imaging parameters of the reticuloendothelial system on FDG PET/CT, comprising BM SUV, BLR, spleen SUV, and SLR, and evaluated whether these parameters showed significant correlations with CD4, CD8, and CD163 cell infiltration and IL-6 expression in gastric cancer tissue. Along with FDG uptake of the BM and spleen, BLR and SLR have also been proposed as imaging parameters of the reticuloendothelial system on FDG PET/CT in previous studies [31,32,33]. As the liver has been generally used as a reference organ in the interpretation of FDG PET/CT images, correction by FDG uptake of the liver was expected to reduce inter-individual variations when compared with BM SUV and spleen SUV [33,34]. The results of our analysis revealed that only the BLR and SLR were significantly associated with CD163 macrophage and CD8 T cell infiltration in the tumor tissue, respectively. Macrophages in the tumor microenvironment, called tumor-associated macrophages, are mostly M2 macrophages with high CD163 expression [9,16,35]. M2 macrophages are known to contribute significantly to the progression and metastasis of gastric cancer, thereby affecting the clinical outcomes of patients [9,35]. Tumor-associated macrophages could be converted from BM-derived macrophages, and the increased metabolic activity of BM-derived macrophages was suggested to be a possible causative factor for increased FDG uptake of BM [20,36]. In a recent study, an increased number of BM-derived tumor-associated macrophages at the tumor invasive margin of hepatic metastasis was found to be significantly associated with worse prognosis in patients with gastric cancer [37]. CD8 T cells are cytotoxic T lymphocytes that can be activated in the spleen [38], which might explain the link between the metabolic activity of the spleen and T lymphocyte infiltration in the tumor tissue. CD8 T lymphocytes are considered to have the capability to destroy cancer cells, and several studies have shown that a high density of CD8 T cell infiltration in tumor tissue was associated with better survival in patients with gastric cancer [1,39]. In contrast, another study revealed that increased CD8 T cell density in the tumor tissue was associated with increased PD-L1 expression in cancer cells, and high intra-tumoral CD8 T cell density was associated with worse prognosis [8]. Therefore, the effects of CD8 T cell immunity in gastric cancer are controversial and might depend on the tumor immune microenvironment [8,40,41]. Considering the significant roles of CD163 macrophages and CD8 T cells in the tumor microenvironment, BLR and SLR might be potential imaging parameters that could help identify the condition of the tumor immune microenvironment in patients with gastric cancer. 

In previous studies, both the BLR and SLR were found to have significant correlations with serum inflammatory markers and were significant predictors of survival in patients with head and neck cancer, non-small cell lung cancer, breast cancer, cholangiocarcinoma, colorectal cancer, uterine cervical cancer, melanoma, and lymphoma, showing consistent results of worse survival in patients with high BM and spleen FDG uptake [14,18,21,26,27,29,31,32,42,43,44,45]. In patients with gastric cancer, only two studies have investigated the prognostic significance of FDG uptake of the reticuloendothelial system in the literature [24,25]. Each study measured BLR [24] and SLR [25] and demonstrated a significant association of BLR and SLR with RFS. Similarly, our results also showed that the BLR and SLR were significant independent predictors of RFS after adjusting for age, sex, TNM stage, and tumor SUV, whereas the BM SUV and spleen SUV failed to show statistical significance. Considering the significant associations of BLR and SLR with both the tumor immune microenvironment and RFS, BLR and SLR could be more appropriate parameters for estimating host immune response to cancer tissue than BM SUV and spleen SUV, respectively, in patients with gastric cancer. However, although both BLR and SLR were significantly associated with RFS, they might reflect different aspects of the host inflammatory response to cancer cells as both imaging parameters showed significant relationships with different immune cell densities in the tumor tissue. In a recent study of non-small cell lung cancer patients, SLR was significantly associated with progression-free survival only in a patient cohort receiving immunotherapy targeting PD-1 [26]. In contrast, BLR was significantly associated with progression-free survival only in a patient cohort receiving chemotherapy [26]. Considering that activated CD8 T cells in the spleen also expressed increased expression of PD-1, SLR might be a more suitable parameter for estimating prognosis in patients who received immunotherapy [38,45]. Further studies are required to establish the proper clinical use of BLR and SLR according to the condition of the tumor immune microenvironment and treatment modalities. 

In the present study, the risk of recurrence after curative surgery could be further predicted using a combination of TNM stage and FDG uptake in the BM and spleen. None of the patients with TNM stage I–II and low metabolic activity of both the BM and spleen experienced cancer recurrence, while cancer recurrence was found in 81.8% of patients with TNM stage III and high metabolic activity of both the BM and spleen. Therefore, in patients with advanced stage gastric cancer who show increased BLR and SLR on staging PET/CT images, an aggressive management strategy would be needed after curative surgery. Furthermore, these results indicate the crucial roles of the host immune response in predicting recurrence risk in gastric cancer, as well as the biological features of cancer cells [1,16,23]. As demonstrated in previous studies on pancreatic cancer and non-small cell lung cancer, incorporating both tumor features and the host immune response could improve the prediction of clinical outcomes when compared solely with the tumor features [20,46]. 

The current study has several limitations. First, the study was retrospectively performed at a single medical center; therefore, there might be some degree of selection bias. Second, because the number of enrolled patients and patients with events was small, the general application of our results might be limited, and further studies with a larger patient cohort are needed to verify the results. Finally, further animal studies and flow cytometry analysis are necessary to elucidate the underlying mechanisms between FDG uptake of the reticuloendothelial system and immunohistochemical results.

## 5. Conclusions

FDG uptake of the reticuloendothelial system on PET/CT was significantly associated with the tumor immune microenvironment and RFS in patients with gastric cancer. In the correlation analysis, BLR and SLR showed significant positive correlations with the degree of CD163 cell and CD8 cell infiltration in the tumor tissue, respectively, and both BLR and SLR were significant predictors of RFS after adjusting for age, sex, TNM stage, and tumor SUV. None of the TNM stage I–II patients with low BLR and SLR experienced cancer recurrence, whereas recurrence was found in 81.8% of stage III patients with high BLR and SLR. Both BLR and SLR might be potential imaging biomarkers that could provide information regarding the tumor immune microenvironment and recurrence risk in patients with gastric cancer.

## Figures and Tables

**Table 1:** Characteristics of patients (n = 70).

Characteristic		No. of Patients (%)	Median (Interquartile Range)
Age (years)			60 (52–72)
Sex	Men	39 (55.7%)	
	Women	31 (44.3%)	
Tumor location	Upper	8 (11.4%)	
	Middle	28 (40.0%)	
	Lower	34 (48.6%)	
WHO histopathological classification	Papillary/tubular types	44 (62.9%)	
	Mucinous/signet ring cells and other poorly cohesive types	26 (37.1%)	
Lauren classification	Intestinal type	30 (42.9%)	
	Diffuse/indeterminate types	40 (57.1%)	
T stage	T1–T2 stage	26 (37.1%)	
	T2–T4 stage	44 (62.9%)	
N stage	N0 stage	29 (41.4%)	
	N1–N3 stage	41 (58.6%)	
TNM stage	Stage I	18 (25.7%)	
	Stage II	19 (27.1%)	
	Stage III	33 (47.1%)	
Adjuvant chemotherapy	Yes	42 (60.0%)	
	No	28 (40.0%)	
CD4 cell infiltration	Grade 0	16 (22.9%)	
	Grade 1	25 (35.7%)	
	Grade 2	18 (25.7%)	
	Grade 3	11 (15.7%)	
CD8 cell infiltration	Grade 0	12 (17.1%)	
	Grade 1	18 (25.7%)	
	Grade 2	23 (32.9%)	
	Grade 3	17 (24.3%)	
CD163 cell infiltration	Grade 0	23 (32.9%)	
	Grade 1	22 (31.4%)	
	Grade 2	13 (18.6%)	
	Grade 3	12 (17.1%)	
IL-6 expression	Grade 0	26 (37.1%)	
	Grade 1	22 (31.4%)	
	Grade 2	17 (24.3%)	
	Grade 3	5 (7.1%)	
FDG PET/CT parameters	Tumor SUV		4.58 (3.32–7.57)
	BM SUV		1.70 (1.48–2.00)
	Spleen SUV		1.66 (1.44–1.81)
	BLR		0.88 (0.75–0.98)
	SLR		0.81 (0.77–0.87)

BLR, bone marrow-to-liver uptake ratio; BM, bone marrow; FDG, 2-deoxy-2-[^18^F]fluoro-D-glucose; IL-6, interleukin-6; PET/CT, positron emission tomography/computed tomography; N stage, node stage; SLR, spleen-to-liver uptake ratio; SUV, standardized uptake value; T stage, tumor stage; TNM stage, tumor, node, and metastasis stage; WHO, World Health Organization.

**Table 2:** Correlation analysis of FDG uptake of the BM and spleen with histopathological results.

Histopathological Factors		BM SUV	*p*-Value	Spleen SUV	*p*-Value	BLR	*p*-Value	SLR	*p*-Value
WHO histopathological classification	Papillary/tubular types	1.69 (1.52–1.97)	0.723 *	1.68 (1.44–1.83)	0.693 *	0.86 (0.75–0.97)	0.879 *	0.82 (0.77–0.87)	0.346 *
	Mucinous/signet ring cells and other poorly cohesive types	1.72 (1.46–2.07)		1.63 (1.52–1.77)		0.88 (0.76–0.98)		0.79 (0.76–0.86)	
Lauren classification	Intestinal type	1.67 (1.48–1.95)	0.618 *	1.55 (1.39–1.85)	0.639 *	0.84 (0.72–0.98)	0.614 *	0.81 (0.76–0.85)	0.753 *
	Diffuse/indeterminate types	1.75 (1.51–2.00)		1.67 (1.52–1.80)		0.88 (0.76–0.97)		0.81 (0.77–0.90)	
TNM stage	Stage I	1.62 (1.48–1.95)	0.170 †	1.70 (1.43–1.81)	0.636 †	0.80 (0.71–0.95)	0.094 †	0.79 (0.75–0.83)	0.080 †
	Stage II	1.68 (1.46–1.90)		1.61 (1.40–1.70)		0.88 (0.75–0.94)		0.81 (0.77–0.89)	
	Stage III	1.79 (1.56–2.02)		1.67 (1.50–1.82)		0.93 (0.77–1.01)		0.83 (0.77–0.91)	
CD4 cell infiltration	Grade 0	1.67 (1.47–1.90)	0.799 †	1.58 (1.42–1.72)	0.818 †	0.80 (0.74–0.91)	0.571 †	0.78 (0.74–0.83)	0.223 †
	Grade 1	1.72 (1.49–2.10)		1.67 (1.49–1.80)		0.93 (0.75–0.98)		0.84 (0.78–0.91)	
	Grade 2	1.67 (1.52–2.07)		1.70 (1.36–88)		0.87 (0.76–0.98)		0.81 (0.77–0.91)	
	Grade 3	1.73 (1.49–1.90)		1.66 (1.53–1.75)		0.84 (0.74–1.01)		0.81 (0.77–0.86)	
CD8 cell infiltration	Grade 0	1.68 (1.53–1.90)	0.943 †	1.58 (1.45–1.64)	0.472 †	0.80 (0.68–0.94)	0.637 †	0.76 (0.71–0.80)	0.016 †
	Grade 1	1.75 (1.46–1.99)		1.72 (1.46–1.88)		0.88 (0.75–0.95)		0.82 (0.78–0.88)	
	Grade 2	1.65 (1.48–2.07)		1.56 (1.43–1.75)		0.89 (0.73–0.98)		0.81 (0.75–0.85)	
	Grade 3	1.79 (1.51–1.96)		1.71 (1.44–1.85)		0.88 (0.79–1.02)		0.84 (0.80–0.94)	
CD163 cell infiltration	Grade 0	1.53 (1.38–1.81)	0.096 †	1.60 (1.44–1.76)	0.474 †	0.79 (0.73–0.92)	0.032 †	0.80 (0.75–0.85)	0.394 †
	Grade 1	1.67 (1.48–1.90)		1.64 (1.35–1.73)		0.84 (0.71–0.94)		0.82 (0.77–0.88)	
	Grade 2	1.95 (1.58–2.18)		1.72 (1.52–1.89)		0.95 (0.76–1.06)		0.81 (0.75–0.86)	
	Grade 3	1.73 (1.58–2.20)		1.72 (1.58–1.78)		0.92 (0.84–1.05)		0.84 (0.79–0.95)	
IL-6 expression	Grade 0	1.66 (1.48–1.94)	0.878 †	1.61 (1.51–1.73)	0.813 †	0.81 (0.75–0.94)	0.743 †	0.80 (0.75–0.88)	0.794 †
	Grade 1	1.75 (1.52–2.00)		1.66 (1.44–1.85)		0.90 (0.75–1.01)		0.82 (0.79–0.91)	
	Grade 2	1.63 (1.46–2.07)		1.69 (1.43–1.80)		0.87 (0.74–0.96)		0.81 (0.77–0.85)	
	Grade 3	1.73 (1.50–2.24)		1.81 (1.43–2.03)		0.89 (0.74–0.97)		0.81 (0.77–0.83)	

All values are expressed in median (interquartile range). * *p*-value for the Mann–Whitney test. † *p*-value for the Kruskal–Wallis test. BLR, bone marrow-to-liver uptake ratio; BM, bone marrow; FDG, 2-deoxy-2-[^18^F]fluoro-D-glucose; IL-6, interleukin-6; SLR, spleen-to-liver uptake ratio; SUV, standardized uptake value; TNM stage, tumor, node, and metastasis stage; WHO, World Health Organization.

**Table 3:** Prognostic significance of the variables for RFS on univariate analysis.

Variables		*p*-Value *	Hazard Ratio (95% Confidence Interval)
Age (<65 years vs. ≥65 years)		0.951	0.99 (0.45–2.16)
Sex (women vs. men)		0.346	1.67 (0.57–4.84)
WHO histopathological classification (papillary/tubular vs. mucinous/signet ring cells and other poorly cohesive types)		0.287	1.52 (0.70–3.29)
Lauren classification (intestinal vs. diffuse/indeterminate)		0.426	1.38 (0.63–3.04)
T stage (T1–T2 vs. T3–T4)		0.003	22.02 (2.98–162.79)
N stage (N0 vs. N1–3)		<0.001	12.92 (3.04–54.88)
TNM stage (stage I–II vs. stage III)		<0.001	15.26 (4.54–51.25)
Adjuvant chemotherapy (yes vs. no)		0.004	4.85 (1.67–14.11)
CD4 cell infiltration (grade 0 vs.)	Grade 1	0.526	1.41 (0.49–4.05)
	Grade 2	0.381	1.63 (0.55–4.88)
	Grade 3	0.206	1.25 (0.29–2.14)
CD8 cell infiltration (grade 0 vs.)	Grade 1	0.848	1.15 (0.29–4.58)
	Grade 2	0.209	2.27 (0.63–8.15)
	Grade 3	0.087	1.90 (0.89–4.15)
CD163 cell infiltration (grade 0 vs.)	Grade 1	0.842	0.90 (0.33–2.49)
	Grade 2	0.146	1.24 (0.41–3.79)
	Grade 3	0.047	2.19 (1.05–5.51)
IL-6 expression (grade 0 vs.)	Grade 1	0.493	1.41 (0.53–3.79)
	Grade 2	0.491	1.45 (0.51–4.12)
	Grade 3	0.042	3.12 (1.07–10.81)
Tumor SUV (<7.53 vs. ≥7.53)		<0.001	4.18 (1.91–9.13)
BM SUV (<1.64 vs. ≥1.64)		0.013	5.79 (1.99–16.88)
Spleen SUV (<1.67 vs. ≥1.67)		0.033	2.41 (1.07–5.42)
BLR (<0.88 vs. ≥0.88)		0.015	2.82 (1.22–6.50)
SLR (<0.84 vs. ≥0.84)		0.040	2.21 (1.02–4.79)

* p-value for Cox proportional hazard regression analysis. BLR, bone marrow-to-liver uptake ratio; BM, bone marrow; IL-6, interleukin-6; N stage, node stage; RFS, recurrence-free survival; SLR, spleen-to-liver uptake ratio; SUV, standardized uptake value; T stage, tumor stage; TNM stage, tumor, node, and metastasis stage; WHO, World Health Organization.

**Table 4:** Prognostic values of FDG PET/CT parameters of the BM and spleen for predicting RFS using multivariate analysis with the addition of age, sex, TNM stage, and tumor SUV as covariates.

Variables	*p*-Value *	Hazard Ratio (95% Confidence Interval)
BM SUV (<1.64 vs. ≥1.64)	0.301	3.58 (0.66–13.78)
Spleen SUV (<1.67 vs. ≥1.67)	0.159	1.75 (0.80–3.80)
BLR (<0.88 vs. ≥0.88)	0.022	3.55 (1.20–10.54)
SLR (<0.84 vs. ≥0.84)	0.049	2.27 (1.01–5.13)

* p-value for Cox proportional hazard regression analysis. BLR, bone marrow-to-liver uptake ratio; BM, bone marrow; FDG, 2-deoxy-2-[18F]fluoro-D-glucose; PET/CT, positron emission tomography/computed tomography; RFS, recurrence-free survival; SLR, spleen-to-liver uptake ratio; SUV, standardized uptake value.

**Table 5:** Recurrence rates of the patient subgroups based on the combination of TNM stage and FDG uptake of BM and spleen.

		TNM Stage	
		Stage I–II	Stage III
**FDG uptake of BM and spleen**	BLR <0.88 and SLR <0.84	0/17 (0.0%)	6/10 (60.0%)
	BLR ≥0.88 and SLR <0.84 Or BLR <0.88 and SLR ≥0.84	0/13 (0.0%)	8/12 (66.7%)
	BLR ≥0.88 and SLR ≥0.84	3/7 (42.9%)	9/11 (81.8%)

BLR, bone marrow-to-liver uptake ratio; BM, bone marrow; FDG, 2-deoxy-2-[18F]fluoro-D-glucose; SLR, spleen-to-liver uptake ratio; TNM stage, tumor, node, and metastasis stage.

## Data Availability

The datasets generated during and/or analyzed during the current study are available from the corresponding authors upon reasonable request.

## Supplementary Materials

The following are available online at https://www.mdpi.com/article/10.3390/life13030771/s1, Table S1: Correlation analysis of FDG uptake of primary tumor lesion, BM, and spleen.

## References

[1] Hibino S., Kawazoe T., Kasahara H., Itoh S., Ishimoto T., Sakata-Yanagimoto M., Taniguchi K. (2021). Inflammation-induced tumorigenesis and metastasis. Int. J. Mol. Sci..

[2] Wen Y., Zhu Y., Zhang C., Yang X., Gao Y., Li M., Yang H., Liu T., Tang H. (2022). Chronic inflammation, cancer development and immunotherapy. Front. Pharmacol..

[3] Kim E.E., Youn H., Kang K.W. (2021). Imaging in tumor immunology. Nucl. Med. Mol. Imaging.

[4] Jaroenlapnopparat A., Bhatia K., Coban S. (2022). Inflammation and gastric cancer. Diseases.

[5] Gu L., Wang M., Cui X., Mo J., Yuan L., Mao F., Zhang K., Ng D.M., Chen P., Wang D. (2020). Clinical significance of peripheral blood-derived inflammation markers in advanced gastric cancer after radical resection. BMC Surg..

[6] Kim M.R., Kim A.S., Choi H.I., Jung J.H., Park J.Y., Ko H.J. (2020). Inflammatory markers for predicting overall survival in gastric cancer patients: A systematic review and meta-analysis. PLoS ONE.

[7] Liu Y., Li C., Lu Y., Liu C., Yang W. (2022). Tumor microenvironment-mediated immune tolerance in development and treatment of gastric cancer. Front. Immunol..

[8] Thompson E.D., Zahurak M., Murphy A., Cornish T., Cuka N., Abdelfatah E., Yang S., Duncan M., Ahuja N., Taube J.M. (2017). Patterns of PD-L1 expression and CD8 T cell infiltration in gastric adenocarcinomas and associated immune stroma. Gut.

[9] Piao H., Fu L., Wang Y., Liu Y., Wang Y., Meng X., Yang D., Xiao X., Zhang J. (2022). A positive feedback loop between gastric cancer cells and tumor-associated macrophage induces malignancy progression. J. Exp. Clin. Cancer Res..

[10] Zhang Z., Zheng B., Chen W., Xiong H., Jiang C. (2021). Accuracy of ^18^F-FDG PET/CT and CECT for primary staging and diagnosis of recurrent gastric cancer: A meta-analysis. Exp. Ther. Med..

[11] Moon S.H., Cho Y.S., Choi J.Y. (2021). KSNM60 in clinical nuclear oncology. Nucl. Med. Mol. Imaging.

[12] Ahn H., Song G.J., Jang S.H., Lee H.J., Lee M.S., Lee J.H., Oh M.H., Jeong G.C., Lee S.M., Lee J.W. (2022). Relationship of FDG PET/CT textural features with the tumor microenvironment and recurrence risks in patients with advanced gastric cancers. Cancers.

[13] Lee S.M., Lee J.W., Lee J.H., Jo I.Y., Jang S.J. (2022). Prognostic Value of Dual-Time-Point [^18^F]FDG PET/CT for predicting distant metastasis after treatment in patients with non-small cell lung cancer. J. Pers. Med..

[14] De Jaeghere E.A., Laloo F., Lippens L., Van Bockstal M., De Man K., Naert E., Van Dorpe J., Van de Vijver K., Tummers P., Makar A. (2020). Splenic ^18^F-FDG uptake on baseline PET/CT is associated with oncological outcomes and tumor immune state in uterine cervical cancer. Gynecol. Oncol..

[15] Bural G.G., Torigian D.A., Chen W., Houseni M., Basu S., Alavi A. (2010). Increased 18F-FDG uptake within the reticuloendothelial system in patients with active lung cancer on PET imaging may indicate activation of the systemic immune response. Hell. J. Nucl. Med..

[16] Parker C.C., Lapi S.E. (2021). Positron emission tomography imaging of macrophages in cancer. Cancers.

[17] Kashimura M. (2020). The human spleen as the center of the blood defense system. Int. J. Hematol..

[18] Lee J.H., Lee H.S., Kim S., Park E.J., Baik S.H., Jeon T.J., Lee K.Y., Ryu Y.H., Kang J. (2021). Prognostic significance of bone marrow and spleen ^18^F-FDG uptake in patients with colorectal cancer. Sci. Rep..

[19] Ishibashi M., Norikane T., Yamamoto Y., Imajo M., Takami Y., Mitamura K., Tanaka T., Tsuruta T., Kanenishi K., Nishiyama Y. (2022). Correlation of bone marrow 2-deoxy-2-[18F]fluoro-D-glucose uptake with systemic inflammation in patients with newly diagnosed endometrial cancer. Nucl. Med. Commun..

[20] Lee J.W., Park S.H., Ahn H., Lee S.M., Jang S.J. (2021). Predicting survival in patients with pancreatic cancer by integrating bone marrow FDG uptake and radiomic features of primary tumor in PET/CT. Cancers.

[21] Kim S.Y., Moon C.M., Yoon H.J., Kim B.S., Lim J.Y., Kim T.O., Choe A.R., Tae C.H., Kim S.E., Jung H.K. (2019). Diffuse splenic FDG uptake is predictive of clinical outcomes in patients with rectal cancer. Sci. Rep..

[22] Seban R.D., Rouzier R., Latouche A., Deleval N., Guinebretiere J.M., Buvat I., Bidard F.C., Champion L. (2021). Total metabolic tumor volume and spleen metabolism on baseline [^18^F]-FDG PET/CT as independent prognostic biomarkers of recurrence in resected breast cancer. Eur. J. Nucl. Med. Mol. Imaging.

[23] Shimura K., Mabuchi S., Komura N., Yokoi E., Kozasa K., Sasano T., Kawano M., Matsumoto Y., Watabe T., Kodama M. (2021). Prognostic significance of bone marrow FDG uptake in patients with gynecological cancer. Sci. Rep..

[24] Lee J.W., Lee M.S., Chung I.K., Son M.W., Cho Y.S., Lee S.M. (2017). Clinical implication of FDG uptake of bone marrow on PET/CT in gastric cancer patients with surgical resection. World J. Gastroenterol..

[25] Yoon H.J., Kim B.S., Moon C.M., Yoo J., Lee K.E., Kim Y. (2018). Prognostic value of diffuse splenic FDG uptake on PET/CT in patients with gastric cancer. PLoS ONE.

[26] Seban R.D., Assié J.B., Giroux-Leprieur E., Massiani M.A., Bonardel G., Chouaid C., Deleval N., Richard C., Mezquita L., Girard N. (2021). Prognostic value of inflammatory response biomarkers using peripheral blood and [18F]-FDG PET/CT in advanced NSCLC patients treated with first-line chemo- or immunotherapy. Lung Cancer.

[27] Lee J.W., Lee S.C., Kim H.J., Lee S.M. (2017). Prognostic value of bone marrow (18)F-FDG uptake on PET/CT in lymphoma patients with negative bone marrow involvement. Hell. J. Nucl. Med..

[28] Elstrom R.L., Tsai D.E., Vergilio J.A., Downs L.H., Alavi A., Schuster S.J. (2004). Enhanced marrow [^18^F]fluorodeoxyglucose uptake related to myeloid hyperplasia in Hodgkin’s lymphoma can simulate lymphoma involvement in marrow. Clin. Lymphoma.

[29] Lee J.W., Ban M.J., Park J.H., Lee S.M. (2019). Effect of F-18 fluorodeoxyglucose uptake by bone marrow on the prognosis of head and neck squamous cell carcinoma. J. Clin. Med..

[30] Pak K., Kim S.J., Kim I.J., Kim D.U., Kim K., Kim H. (2013). Impact of cytokines on diffuse splenic 18F-fluorodeoxyglucose uptake during positron emission tomography/computed tomography. Nucl. Med. Commun..

[31] Pak K., Kim S.J., Kim I.J., Kim D.U., Kim K., Kim H., Kim S.J. (2014). Splenic FDG uptake predicts poor prognosis in patients with unresectable cholangiocarcinoma. Nuklearmedizin.

[32] Lee J.W., Na J.O., Kang D.Y., Lee S.Y., Lee S.M. (2017). Prognostic significance of FDG uptake of bone marrow on PET/CT in patients with non-small-cell lung cancer after curative surgical resection. Clin. Lung. Cancer..

[33] Inoue K., Goto R., Okada K., Kinomura S., Fukuda H. (2009). A bone marrow F-18 FDG uptake exceeding the liver uptake may indicate bone marrow hyperactivity. Ann. Nucl. Med..

[34] Yoo I.D., Lee S.M., Lee J.W., Oh J.E., Cho Y.J., Shin H.S. (2017). The influence of adipose tissue volume can significantly affect the metabolic activity of reference organs in (18)F-FDG PET/CT studies of a normal healthy population. Hell. J. Nucl. Med..

[35] Li W., Zhang X., Wu F., Zhou Y., Bao Z., Li H., Zheng P., Zhao S. (2019). Gastric cancer-derived mesenchymal stromal cells trigger M2 macrophage polarization that promotes metastasis and EMT in gastric cancer. Cell Death Dis..

[36] Iwamoto C., Ohuchida K., Shinkawa T., Okuda S., Otsubo Y., Okumura T., Sagara A., Koikawa K., Ando Y., Shindo K. (2021). Bone marrow-derived macrophages converted into cancer-associated fibroblast-like cells promote pancreatic cancer progression. Cancer Lett..

[37] Wang Z., Dong Z., Zhao G., Ni B., Zhang Z.Z. (2022). Prognostic role of myeloid-derived tumor-associated macrophages at the tumor invasive margin in gastric cancer with liver metastasis (GCLM): A single-center retrospective study. J. Gastrointest. Oncol..

[38] Wang Z., Chen T., Lin W., Zheng W., Chen J., Huang F., Xie X. (2020). Functional tumor specific CD8 + T cells in spleen express a high level of PD-1. Int. Immunopharmacol..

[39] Zhang N., Cao M., Duan Y., Bai H., Li X., Wang Y. (2020). Prognostic role of tumor-infiltrating lymphocytes in gastric cancer: A meta-analysis and experimental validation. Arch. Med. Sci..

[40] Wei M., Shen D., Mulmi Shrestha S., Liu J., Zhang J., Yin Y. (2018). The progress of T cell immunity related to prognosis in gastric cancer. Biomed. Res. Int..

[41] Rahn S., Krüger S., Röcken C., Helm O., Sebens S. (2019). Response to: ‘Patterns of PD-L1 expression and CD8 T cell infiltration in gastric adenocarcinomas and associated immune stroma’. Gut.

[42] Kim C.G., Hwang S.H., Kim K.H., Yoon H.I., Shim H.S., Lee J.H., Han Y., Ahn B.C., Hong M.H., Kim H.R. (2022). Predicting treatment outcomes using (18)F-FDG PET biomarkers in patients with non-small-cell lung cancer receiving chemoimmunotherapy. Ther. Adv. Med. Oncol..

[43] Lee J.W., Jeon S., Mun S.T., Lee S.M. (2017). Prognostic value of fluorine-18 fluorodeoxyglucose uptake of bone marrow on positron emission tomography/computed tomography for prediction of disease progression in cervical cancer. Int. J. Gynecol. Cancer.

[44] Lee J.W., Kim S.Y., Han S.W., Lee J.E., Lee H.J., Heo N.H., Lee S.M. (2020). [(18)F]FDG uptake of bone marrow on PET/CT for predicting distant recurrence in breast cancer patients after surgical resection. EJNMMI Res..

[45] Wong A., Callahan J., Keyaerts M., Neyns B., Mangana J., Aberle S., Herschtal A., Fullerton S., Milne D., Iravani A. (2020). (18)F-FDG PET/CT based spleen to liver ratio associates with clinical outcome to ipilimumab in patients with metastatic melanoma. Cancer Imaging.

[46] Mattonen S.A., Davidzon G.A., Benson J., Leung A.N.C., Vasanawala M., Horng G., Shrager J.B., Napel S., Nair V.S. (2019). Bone marrow and tumor radiomics at (18)F-FDG PET/CT: Impact on outcome prediction in non-small cell lung cancer. Radiology.

