# Supplementary material for: Relationship of FDG Uptake of the Reticuloendothelial System with Tumor Immune Microenvironment and Prognosis in Patients with Gastric Cancer
Source: Life (Basel). 2023 Mar 13;13(3):771. doi: 10.3390/life13030771 (PMC10053773; doi:10.3390/life13030771)
Supplement: Supplementary file 1 [file life-13-00771-s001.zip › life-2209212-supplementary.pdf]

Article Title: **Relationship of FDG uptake of the reticuloendothelial system with tumor immune microenvironment and prognosis in patients with gastric cancer**

Authors: Hyein Ahn, Geum Jong Song, Moon-Soo Lee, Ji-Hye Lee, Si-Hyong Jang, Mee-Hye Oh, Jong Hyuk Yun, Sang Mi Lee, Jeong Won Lee

Table S1. Correlation analysis of FDG uptake of primary tumor lesion, BM, and spleen.

		Tumor SUV	BM SUV	Spleen SUV	BLR	SLR
Tumor SUV	Correlation coefficient (95% CI)	1.00	0.402 (0.184–0.582)	0.296 (0.066–0.497)	0.374 (0.152–0.560)	0.474 (0.268–0.638)
	P-value	-	<0.001	0.013	0.014	<0.001
BM SUV	Correlation coefficient (95% CI)	0.402 (0.184–0.582)	1.00	0.616 (0.446–0.744)	0.641 (0.513–0.731)	0.407 (0.190–0.586)
	P-value	<0.001	-	<0.001	<0.001	<0.001
Spleen SUV	Correlation coefficient (95% CI)	0.296 (0.066–0.497)	0.616 (0.446–0.744)	1.00	0.133 (-0.105–0.357)	0.622 (0.437–0.774)
	P-value	0.013	<0.001	-	0.272	<0.001
BLR	Correlation coefficient (95% CI)	0.374 (0.152–0.560)	0.641 (0.513–0.731)	0.133 (-0.105–0.357)	1.00	0.622 (0.437–0.774)
	P-value	0.014	<0.001	0.272	-	<0.001
SLR	Correlation coefficient (95% CI)	0.474 (0.268–0.638)	0.407 (0.190–0.586)	0.622 (0.437–0.774)	0.622 (0.437–0.774)	1.00
	P-value	<0.001	<0.001	<0.001	<0.001	-

BLR, bone marrow-to-liver uptake ratio; BM, bone marrow; CI, confidence interval; FDG, 2-deoxy-2-[^{18}F]fluoro-D-glucose; SLR, spleen-to-liver uptake ratio; SUV, standardized uptake value
